# 278 Trends in Clostridioides difficile Diagnostic Testing Methods in U.S. Acute-Care Hospitals

**DOI:** 10.1017/ash.2026.10450

**Published:** 2026-06-23

**Authors:** Courtney Takats, Gregory Putzel, Alice Tillman, Julian McWilliams, Magdalena Podkowik, Michael Phillips, Alejandro Pironti, Bo Shopsin, Sarah Hochman

**Affiliations:** 1 NYU Langone Health; 2 NYU Langone; 3 NYU School of Medicine; 4 NYU Langone Medical Center

## Abstract

**Background:** Staphylococcus aureus is a leading cause of healthcare-associated infections and is associated with high mortality. While decolonization has been effective in reducing methicillin-resistant S. aureus (MRSA) infections in adult and neonatal intensive care units (NICUs), little is known about the impact of decolonization on transmission of S. aureus. Here, we evaluated whether weekly screening and targeted decolonization reduce genomically defined transmission of S. aureus in a NICU. **Methods:** Infants admitted in 2022-2024 to the NICU at Tisch Hospital received weekly screening cultures of the nares, axilla and groin for MRSA and methicillin-susceptible S. aureus (MSSA). Colonized infants received topical decolonization with chlorhexidine 2% bathing and nasal, buttock, and umbilical mupirocin, with frequency and duration of decolonization based on postmenstrual age (gestational plus chronological age). Genome sequencing was used to identify transmission events, defined as genetically linked S. aureus isolates with <20 single nucleotide variants identified in two infants with overlapping stays. Transmission risk was analyzed using time-to-event (TTE) analyses, and the absolute risk reduction and number needed to treat (NNT) were estimated. **Results:** Among 1,597 screened infants, 188 (11.8%) were colonized with S. aureus (85.6% MSSA; 14.4% MRSA). Colonized infants had a median NICU length of stay of 96 days, and 39 (20.7%) were involved in at least one transmission event. Of these infants, 84.6% received full decolonization. Across the cohort, 389 conversions from colonization negative to positive were observed, including 97 recolonization events; 55 (56.7%) of these infants became colonized ? 3 times during their stay, indicating sustained exposure and transmission pressure. TTE modeling predicted a 30-day transmission risk of 0.6% if all patients were decolonized, and 8.9% risk if no patients were decolonized. This corresponds to a 30-day absolute risk reduction for transmission of 8.2% and an NNT of 12; decolonizing 12 infants prevented one S. aureus transmission. **Conclusions:** Genomically informed modeling indicates that weekly screening and targeted decolonization reduce S. aureus transmission in the NICU. Frequent recolonization supports the importance of weekly S. aureus screening and suggests that decolonization prevents infection partly by limiting spread to susceptible hosts, with implications for the spread of resistant strains. Future analyses incorporating time-varying eligibility and exposure will further refine these transmission estimates.

